# Reusable Lignin‐Derived Free‐Standing Carbon Nanofiber Sulfur Hosts *via* Carbonization Engineering for Sustainable Lithium–Sulfur Batteries

**DOI:** 10.1002/gch2.70157

**Published:** 2026-09-08

**Authors:** Kshitij Mahesh Kakad, Qingping Wu, Seyedrashid Mirmasoomi, Sijia Cao, Johannes Schenk, Marius Hermesdorf, Vikram Raghuraman, Liqiang Lu, Martin Oschatz, Yan Lu

**Affiliations:** ^1^ Institute of Electrochemical Energy Storage Helmholtz‐Zentrum Berlin für Materialien und Energie GmbH Berlin Germany; ^2^ Institute for Technical and Environmental Chemistry Friedrich‐Schiller‐Universität Jena Jena Germany; ^3^ Helmholtz Institute for Polymers in Energy Applications (HIPOLE Jena) Jena Germany; ^4^ Center for Energy and Environmental Chemistry Jena (CEEC Jena) Friedrich‐Schiller‐Universität Jena Jena Germany

**Keywords:** carbonization engineering, electrode recycling, free‐standing electrodes, kraft lignin, lithium–sulfur batteries

## Abstract

As an abundant bio‐based resource, lignin can be converted to functional carbon materials, thereby providing a sustainable pathway toward free‐standing sulfur cathode design. However, rational carbonization engineering remains essential for optimizing sulfur utilization and polysulfide conversion behavior in lithium–sulfur batteries. In this work, lignin valorization and free‐standing electrode design are integrated through electrospinning‐assisted fabrication of nanofibers, followed by high temperature carbonization to produce flexible and reusable sulfur hosts. The interconnected fibrous architecture facilitates homogeneous sulfur distribution, while promoting polysulfide confinement and electrochemical stability under high sulfur loading and lean electrolyte. At a sulfur loading of 3.3 mg cm^−2^, the electrode affords a high initial capacity of 1300 mAh g^−1^. At an electrolyte content of 5.5 µL_E_ mg_S_^−1^, the coin cells are shown to cycle steadily with capacity retentions of 82.9% after 100 cycles at 0.2 C. Furthermore, the reusability of these electrodes is confirmed via successful recovery, with the recovered electrode demonstrating stable cycling at 1 C, while retaining 79.3% of its initial capacity after 150 cycles. This research thus confirms the potential of lignin‐derived, green, and cost‐effective approach of synthesizing free‐standing electrodes as efficient sulfur host materials with reliable and steady battery cycling performance.

## Graphical abstract

Lignin has been valorized into free‐standing sulfur cathodes via a sustainable electrospinning‐assisted nanofiber synthesis. The flexible electrode allows homogeneous sulfur distribution, promotes polysulfide confinement, and can be effectively reused in new coin cells. The recovered electrode can cycle stably with 80% capacity retention over 150 cycles at 1 C, with uniform distribution of carbon and sulfur in the cycled electrode.

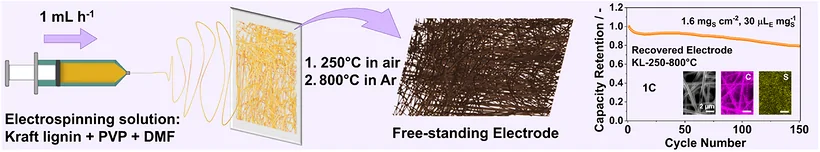

## 1 Introduction

Lithium–sulfur batteries (LSBs) have emerged as promising next‐generation energy storage systems because of the high theoretical specific capacity of sulfur as cathode active material (1675 mAh g^−1^) coupled with lithium metal anodes (3860 mAh g^−1^), far exceeding that of conventional lithium‐ion batteries (LIBs) [1, 2]. Further benefits of substituting the established cathode with sulfur include its widespread availability, safety, minimal environmental impact, and cost‐effectiveness [3, 4]. Despite such advantages, LSBs encounter several fundamental limitations, including the poor conductivity of sulfur and Li_2_S, pronounced volume changes during cycling, and dissolution of lithium polysulfides (LiPSs) in ether‐based electrolytes [5, 6, 7]. These issues can cause electrode degradation, capacity fading, LiPS shuttle effect, and parasitic reactions at the lithium anode, impeding the overall electrochemical performance [5, 6, 7, 8]. The loss of polysulfides can be suppressed by hybridizing sulfur with nanocarbons, such as Ketjenblack, carbon nanotubes, and carbon nanofibers (CNFs), as well as transition metal chalcogenides and pnictogenides, leading to physical and chemical confinement of sulfur, Li_2_S, and intermediates [9, 10, 11, 12, 13, 14, 15, 16].

With carbon being an essential element for developing sulfur host materials, it is crucial to investigate sustainable bio‐based resources for it. Biomass‐derived carbons have already been implemented as electrode materials or functional materials for various battery applications including in LSBs. Several different sources, including woody biomass, agricultural residue, marine plants, municipal waste, as well as human‐ and animal‐based biomass have been used for this purpose [17, 18, 19]. In recent years, lignin has gained considerable attention as a sustainable bio‐based carbon source owing to its economic viability, high molecular weight, rich aromatic structures, and natural abundance owing to it being an extensively underutilized industrial waste from the paper and pulp industry [20, 21, 22, 23]. Lignin is typically thermally utilized to generate heat and electricity but there is huge interest to establish value‐chains for its chemical utilization or the conversion into functional materials. Lignin has already been effectively implemented in LSBs. For instance, incorporating lignin into multi‐walled carbon nanotube (CNT)‐based protection layers can reduce costs and enhance electrochemical performance. Benefitting from strengthened LiPS transport and enhanced lithium‐ion diffusion, this system achieved high initial capacity of 1347 mAh g^−1^ at 0.5 C with a low decay rate of 0.2% per cycle [24]. Lignin has also been implemented as a binder in ultra‐low dosage for enhancing adhesion of the host material and conductive carbon with sulfur and Li_2_S, as well as for inhibiting volumetric variation and shuttle effect, owing to its semi‐rigid structure and three‐dimensional network. Even under an ultra‐low dosage of 2 wt.%, a high initial capacity of 864 mAh g^−1^ was obtained at 0.5 C [25]. The role of the binder is to strengthen the interlocking between sulfur, the host, and the current collector, but as an inactive component, its presence leads to reduced conductivity as well as obstruction of active sites [26, 27]. Thus, it is critical to redirect attention toward self‐standing electrodes that do not require binders or current collectors. Lignin CNFs embedded with MoS_2_ were fabricated via electrospinning, which delivered enhanced redox kinetics and stable cycling, providing a capacity of nearly 610 mAh g^−1^ even after 200 cycles at 1 C [28]. Furthermore, it is critical to investigate the reusability and recyclability of electrode materials in energy storage systems. In this regard, a graphite electrode was successfully recovered from cycled LIBs and reused as a host material for LSBs, thereby obtaining 224 mAh g^−1^ after 100 cycles at 0.1 C, under a sulfur loading of 68 wt.% [29]. Moreover, failure of LSBs due to overcharging was investigated, revealing the durability of the regenerated sulfur cathode from a coin cell that suffered internal short‐circuits, which when reassembled with a new lithium metal anode was still able to cycle stably [30].

One key question in sustainable LSB design is how to convert biomass‐derived carbon precursors into structurally robust, electrochemically active, and regenerable sulfur hosts. Herein, this issue has been addressed by developing reusable lignin‐derived free‐standing CNF sulfur hosts through electrospinning and carbonization engineering. Kraft lignin is employed as a sustainable bio‐based carbon precursor, while electrospinning enables the construction of an interconnected fibrous membrane that can be directly used as a sulfur host without addition of binder and current collector. By tuning the carbonization temperature, the relationship between carbon structure evolution and electrochemical behavior of LSBs is systematically investigated. The optimized electrode carbonized at 800°C (KL‐250‐800°C) delivers balanced polysulfide adsorption, sulfur conversion kinetics, rate capability, and cycling stability, even under high sulfur‐loading and lean‐electrolyte conditions. More importantly, the cycled electrode can be regenerated by replenishing fresh Li_2_S_8_ catholyte, highlighting the structural robustness and recyclability of the free‐standing lignin‐derived host. This work provides a sustainable and recyclable electrode design strategy for high‐performance LSBs.

## 2 Results and Discussion

**FIGURE 1:**
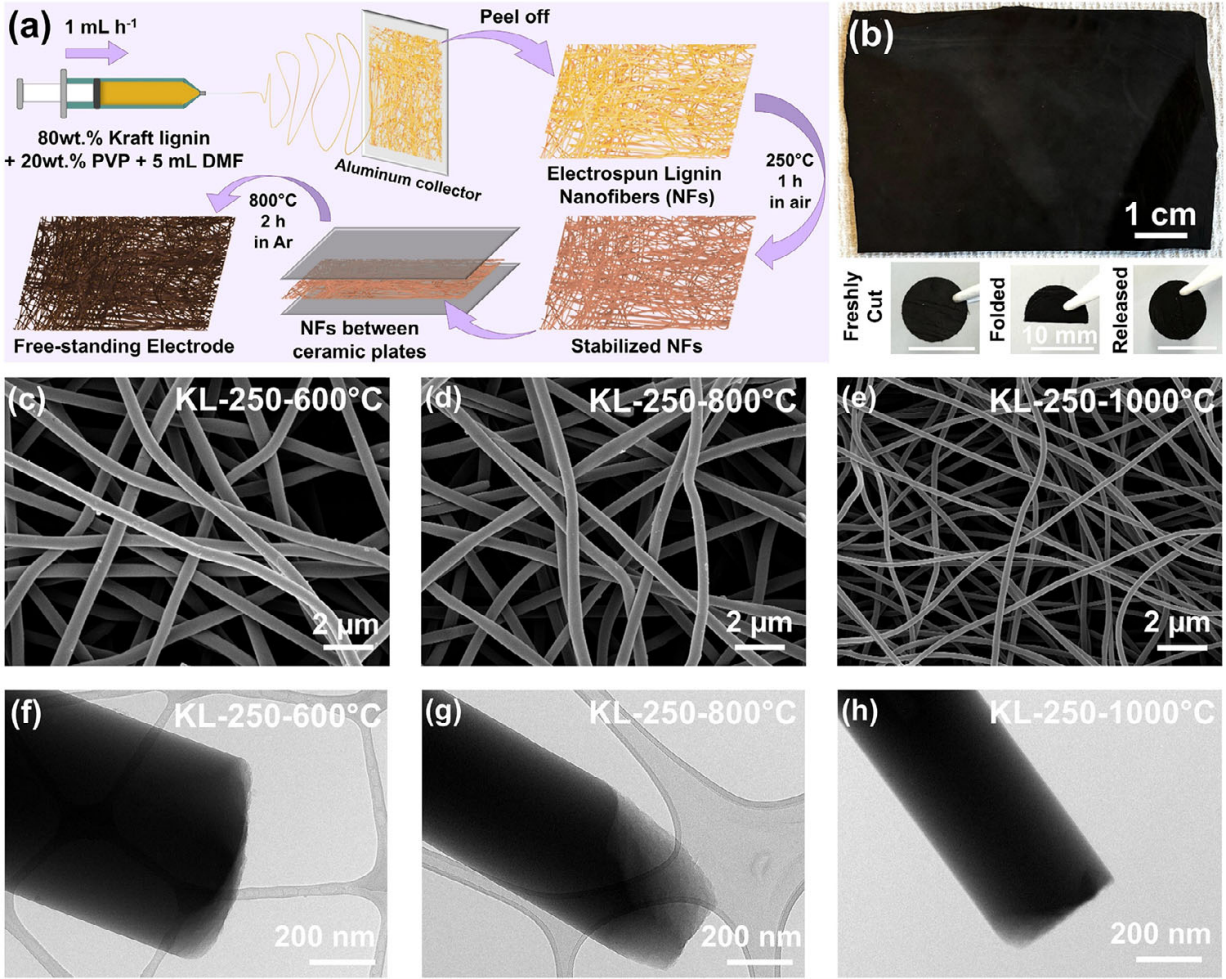
(a) Schematic illustration of the synthesis of nanofibers from Kraft lignin via electrospinning with polyvinylpyrrolidone (PVP) as the copolymer and dimethylformamide (DMF) as the solvent, followed by pre‐oxidation at 250°C and carbonization at 600°C, 800°C, and 1000°C (here exemplarily shown for 800°C). (b) Digital image of the obtained free‐standing electrode after carbonization at 800°C, along with images of the circular 10‐mm electrodes cut out for coin cell applications. Scanning electron microscopy (SEM) images of the samples at three carbonization temperatures (c) KL‐250‐600°C, (d) KL‐250‐800°C, and (e) KL‐250‐1000°C. High‐resolution transmission electron microscopy (HR‐TEM) images of individual fibers obtained at different carbonization temperatures of (f) 600°C, (g) 800°C, and (h) 1000°C.

In this study, Kraft lignin was used as bio‐based carbon precursor (supplied by Mercer Rosenthal). It was processed via the LignoBoost process by precipitation of black liquor from the kraft pulp mill. The weight‐averaged molecular weight of the used lignin is 15 876 g mol^−1^ with dry and ash contents of 59 wt.% and 1.26 wt.%, respectively. As a sustainable method of obtaining nanofibers, electrospinning was employed to prepare lignin‐based nanofibers, as schematized in Figure 1a. Pure lignin does not electrospin on its own due to its comparatively short molecular chains, poor flexibility, as well as complex and irregular molecular structure and arrangement. A straight‐forward method to overcome this issue is the addition of a high‐molecular‐weight copolymer containing long‐chains to enhance molecular chain entanglement [31, 32]. Herein, the electrospinning solution was prepared by dissolving 80 wt.% of lignin and 20 wt.% polyvinylpyrrolidone (PVP, M_w_ = 1 300 000) in 5 mL N,N‐dimethylformamide (DMF, 99.8%), which was drawn into a syringe and attached to the electrospinning system with a needle, flow rate controller, and high‐voltage cable. An aluminum collector was used for fiber deposition and the distance between the collector and the needle was set to 15 cm. The flow rate was maintained at 1 mL h^−1^ and a voltage of ∼9 kV was applied. After the electrospinning was completed, the fibrous mat deposited was peeled off and stabilized by pre‐oxidizing in air at a very low heating rate of 0.5°C min^−1^ to 250°C for 1 h. This is an essential step for enhancing crosslinking and preventing the fibers from melting or merging into one another during the subsequent carbonization step [31, 32]. Next, the stabilized fibers were placed between two ceramic plates and inserted inside a horizontal tube furnace and calcinated at 600°C, 800°C, and 1000°C at a heating rate of 5°C min^−1^ for 2 h under argon flow. This resulted in free‐standing carbon electrodes that could be directly cut into 10‐mm electrode discs and implemented in coin cells for electrochemical studies. A digital image of the carbonized free‐standing electrode is presented in Figure 1b, along with the 10‐mm electrode discs, also highlighting their flexibility and bendability. Thus, the need for binders and conductive materials has been completely eliminated, additionally highlighting the sustainable nature of the electrode preparation process via electrospinning. The samples are labelled based on the pre‐oxidation and the carbonization temperatures. For example, the carbonized nanofibers obtained after pre‐oxidation at 250°C and calcination at 600°C, 800°C, and 1000°C are denoted as KL‐250‐600°C, KL‐250‐800°C, and KL‐250‐1000°C, respectively.

To observe the surface morphology of the CNFs obtained, SEM was performed, and the SEM images are shown in Figure 1c–e for the CNFs carbonized at 600°C, 800°C, and 1000°C, respectively. Digital images of the electrospun and stabilized fibrous mat are shown in Figure S1a,b, and their corresponding high‐resolution scanning electron microscopy (SEM) images in Figure S1c,d, respectively. Additionally, low‐resolution SEM images of the CNFs at different stages of the synthesis process are presented in Figure S2. A uniform fibrous network with long and continuous fibers with low density of defects has been observed for all the three samples, with the fiber morphology being preserved even after high‐temperature treatment. The diameter of the individual fibers is reduced with increasing carbonization temperature. The average diameters for the three samples KL‐250‐600°C, KL‐250‐800°C, and KL‐250‐1000°C were measured to be 547.4 ± 59.4, 497.4 ± 42.9, and 454.5 ± 53.8 nm, respectively. These average values were obtained by measuring 150 fibers from the low‐resolution SEM images using the ImageJ software. The Gaussian distribution of these diameters is shown in Figure S3. The reduction in fiber diameter can be attributed to loss of mass due to more pronounced condensation processes during carbonization at higher temperature. This is additionally confirmed by the Fourier‐transform infrared (FT‐IR) spectra, shown in Figure S4. For the as‐prepared lignin nanofibers and for the nanofibers pre‐oxidized at 250°C, there are clear peaks observed for all the functional groups presents, namely the broad –OH stretch, the asymmetric –CH_2_ stretch, symmetric –CH_2_ stretch, C = O stretch, C–N stretch, and C–H bend at wavenumbers of 3350, 2390, 2850, 1650, 1420, and 1270 cm^−1^ [33, 34]. The peaks at 1510 and 1595 cm^−1^ represent the C = C aromatic stretch, confirming the presence of benzene rings in lignin [35, 36]. The intensity of these peaks is significantly reduced after carbonization at 600°C, and completely disappear at higher temperatures of 800°C and 1000°C, indicating loss of functional groups containing volatile elements such as oxygen and nitrogen, structural ordering and smoothening of the fibers originating from the phase transition of the polymers to carbonized structures, as well as the loss of some carbon as CO_2_ or in the form of other volatile species [37, 38].

To confirm that these nanofibers are completely dense solids and comprise no internal porosities or structuring, high‐resolution transmission electron microscopy (HR‐TEM) images were obtained for the three samples, as shown in Figure 1f–h. The opaqueness of the individual fibers confirms the lack of large internal voids within the fibers themselves. Fiber diameters of the 600°C, 800°C, and 1000°C samples presented in these figures are measured to be 635.7, 436.1, and 397.2 nm, respectively, which differs slightly from the average values from the SEM images, but they fall within the Gaussian distribution of the 150 fibers measured shown in Figure S3. The trend in the fiber diameter is also similar, with the fibers getting thinner with increasing temperature. The low‐resolution TEM images are shown in Figure S5, from which it can be observed that the breakability of the individual fibers synthesized at higher temperatures is greater, indicating enhanced brittleness with increasing calcination temperatures.

To obtain information about the chemical composition of lignin, elemental analysis was conducted at each stage of the synthesis process, that is, the as‐prepared lignin nanofibers, the stabilized fibers, and the free‐standing electrodes calcinated at different temperatures. The results are listed in Table S1. The carbon content in the as‐prepared lignin nanofibers is measured to be 60.76 wt.%, which is lower than that reported for pure Kraft lignin in the literature of nearly 63 wt.%, whereas the nitrogen content is 3.49 wt.%, which is higher than that reported in the literature [39, 40, 41]. These slight variations from the values reported in the literature likely arise from the presence of PVP in the electrospinning solution, which has afforded nanofibers in 80:20 weight ratios of lignin and PVP. In addition, the carbon content is observed to increase whereas the nitrogen, sulfur, and hydrogen content is noted to gradually reduce with increasing calcination temperature, which supplements the expected loss of functional groups from the FT‐IR results and the thinning of the fibers with increasing temperatures.

**FIGURE 2:**
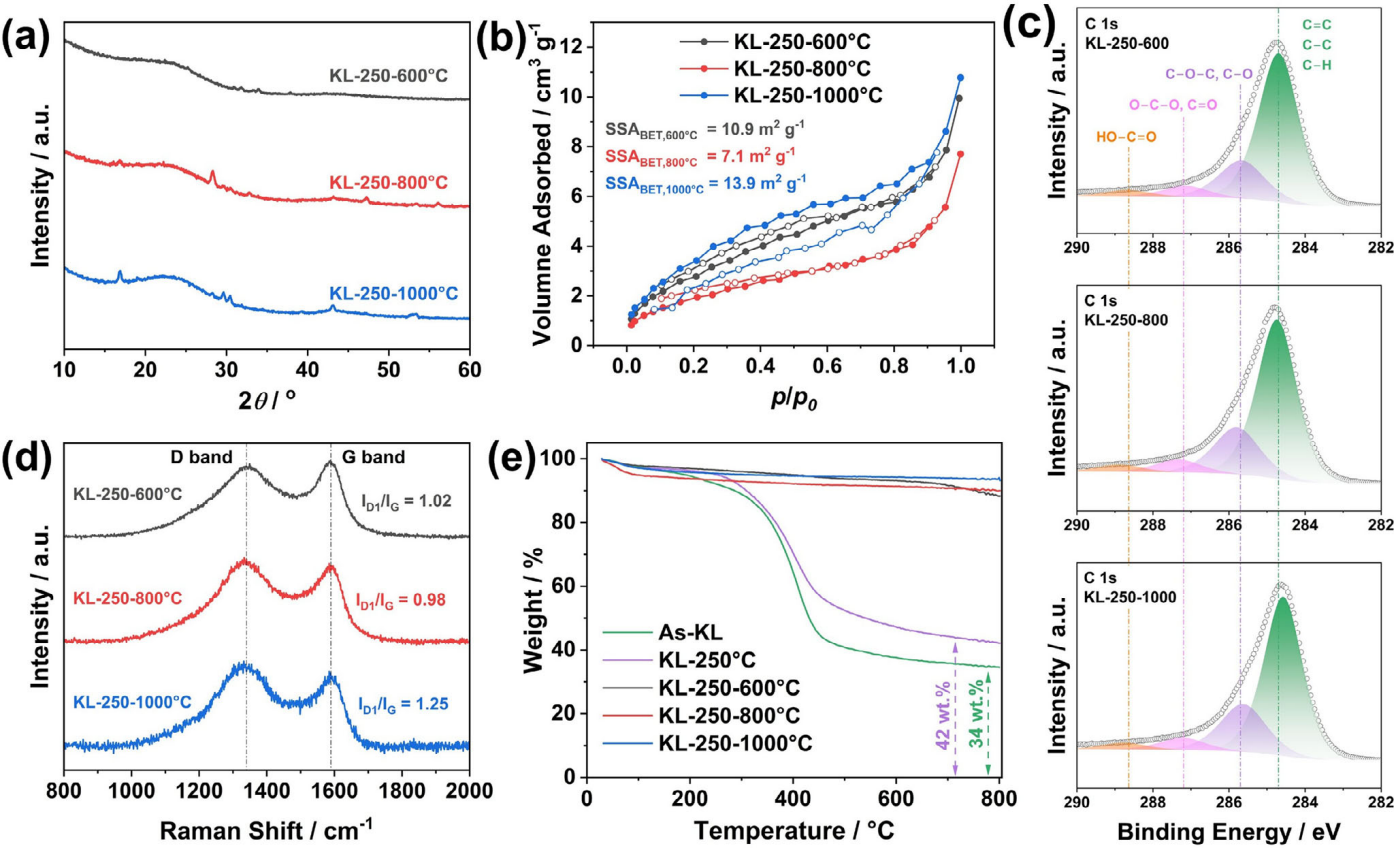
(a) X‐ray diffraction patterns of the KL‐250‐600°C, KL‐250‐800°C, and KL‐250‐1000°C fibers. (b) N_2_ adsorption–desorption isotherms measured at 77 K, (c) C *1s* XPS spectra, (d) Raman spectra, and (e) thermogravimetric analysis curves measured under argon flow of the three fiber samples after carbonization at different temperatures.

To evaluate the impact of carbonization conditions on the carbon nanostructure within lignin‐derived CNFs, X‐ray diffraction (XRD) of the fibers carbonized at different temperatures was conducted, and the measured patterns are shown in Figure 2a. The diffractograms demonstrate majorly amorphous structures with a broad reflex at ∼22°, which represents the (002) reflection for disordered carbon. Some minor signals are observed at 2ϴ values of 16.8°, 28.2°, 29.6°, 30.3°, 43.0°, 47.2°, 53.3°, and 56.0°. These minor reflections could be reasonably attributed to inorganic residues or ash‐derived mineral impurities retained from the lignin precursor after carbonization. Therefore, the XRD results indicate that the obtained CNFs are mainly composed of disordered carbon with limited local ordering, while trace inorganic residual species may coexist within the carbonized fibers. To analyze the pore structure and the specific surface area, N_2_ adsorption–desorption isotherms were obtained, which are shown in Figure 2b, including the corresponding values of the calculated Brunauer−Emmett−Teller specific surface areas. Based on these results, all the samples can be considered to be non‐porous. It must be noted that this non‐porosity relates to the intrinsic nanoporosity of the fibers themselves. The gaps between the individual fibers, which are not detectable via N_2_ physisorption, are essential for the physical confinement of sulfur. This can also be additionally supported by the corresponding pore size distribution and cumulative pore volumes shown in Figure S6a,b, which indicates a more random distribution of the pore widths, ranging from 1–8 nm with no clear overarching peaks and very low cumulative pore volumes.

The surface chemical state and the surface composition of the fibers were tested using X‐ray photoelectron spectroscopy (XPS). The high‐resolution C *1s* spectra and the corresponding deconvoluted peaks for the three samples are shown in Figure 2c. The peak at 284.7 eV represents the sp^2^ hybridized graphitic carbon bonds as well as sp^3^ carbon contributions (C = C, C−C, and C−H), the peak at 285.7 eV reflects the weakly oxidized carbon species (C−O and C−O−C), the one at 287.2 eV represents the highly oxidized carbons, that is, the aldehyde, keto, and carbonyl groups (C = O, O−C−O), and the peak at 288.6 eV refers to the carboxyl groups (−COOH) [42, 43, 44]. For all the samples, the atomic percentage of carbon was noted to be ∼95%, highlighting a predominant peak for graphitic carbon as well as to possible amorphous carbon. Thus, as a next step, Raman spectra for the three samples were measured to study the extent of graphitization and disorder among the different samples, and the obtained spectra are shown in Figure 2d. The peak at 1336 cm^−1^ is the D band representing the disordered carbon (sp^2^ hybridized C−C in‐plane breathing mode, representing structural defects and bent planes) and that at 1584 cm^−1^ is the G band, which originates from sp^2^ hybridized C−C in‐plane stretching mode from carbon atoms in chains or rings [45]. An essential parameter that can be obtained from the Raman spectra is the ratios of the intensity of D and G bands (I_D_/I_G_) to study the degree of carbonization due to the formation of disordered sp^2^ carbon rings of disorder present. To adequate analyze this information, it is essential to fit the different peaks contributing to this curve. The disorders themselves can be split into three types. The D1 band at nearly 1325 cm^−1^ represents the graphitic lattice disorders referring to the graphene layer edges, the D2 band around 1200 cm^−1^ refers to the graphitic lattice disorders as well as any ionic impurities, and the A band at about 1500 cm^−1^ is attributed to amorphous carbon [45]. The curves are fitted using OriginPro following the Gaussian distribution, and the fitted data are shown in Figure S7. These I_D_/I_G_ values stand at 1.02, 0.98, and 1.25 for the 600°C, 800°C, and 1000°C samples, respectively, indicating first comparable carbonization decrease followed by an expected increase with increasing carbonization temperatures. This demonstrates that the nanostructures of all samples are quite similar in this temperature range, and with increasing temperatures, more graphitic domains are being formed, which are the source of the increase in the D band intensity. As shown in the TEM images in Figure S5, the brittleness and breakability of the fibers is higher after carbonization at 1000°C, signifying disordered surfaces and increase in amorphous nature of carbon.

For additionally confirming the loss of the functional groups as evidenced by the FT‐IR spectra (Figure S4), thermogravimetric analysis (TGA) was performed for the as‐prepared, stabilized, and carbonized samples in argon atmosphere, and the results are shown in Figure 2e. All samples show a minor weight loss at around 100°C, which refers to the loss of absorbed moisture. The further mass loss until 300°C relates to the condensation and decomposition of different components in lignin. Generally, the process of carbonization under inert atmosphere progresses with the breakage of functional groups that contain oxygen, leading to a reduction in oxygen amount and increase in carbon content. The as‐prepared and the pre‐oxidized samples exhibit their major mass losses from 300°C to 500°C, representing the main decomposition stage and the loss of the remaining functional groups in amounts of 34 and 42 wt.%, respectively. For the three carbonized samples, the residual mass after TGA mainly corresponds to the carbonaceous framework. For the 600°C, 800°C, and 1000°C, the remaining masses after TGA until 800°C are 88.3 wt.%, 90.0 wt.%, and 93.2 wt.%, respectively, confirming increase in carbon content with increasing temperatures. Furthermore, for the 600°C sample, residual carbonization is observed to take place between 600°C and 800°C, which refers to its further loss in mass in this range [46].

**FIGURE 3:**
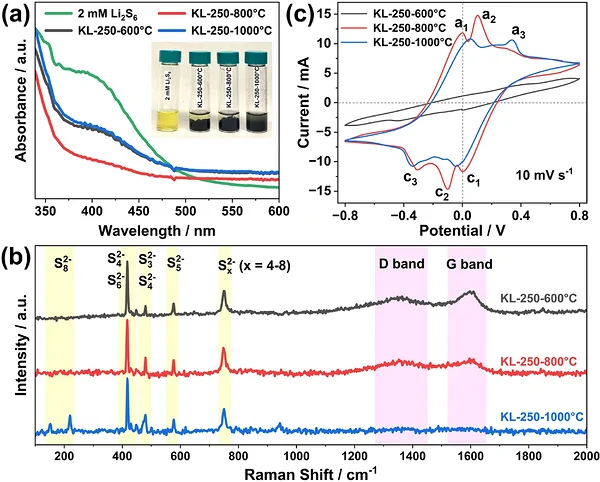
(a) UV–vis absorption spectra of the supernatant of the adsorption test using 2 mL, 2 mM Li_2_S_6_ as the representative of the polysulfides. Inset: Digital image of the polysulfide adsorption test. (b) Raman spectra of the three carbonized fiber samples after the LiPS adsorption test. (c) Cyclic voltammograms of symmetric cells assembled for the free‐standing electrodes carbonized at different temperatures at 10 mV s^−1^ from −0.8 V to 0.8 V.

To study the affinity between the lignin fibers synthesized at different temperatures and LiPSs, polysulfide adsorption test was carried out. Herein, 2 mM Li_2_S_6_ in equal volumetric ratios of 1,3‐dioxolane (DOL) and 1,2‐dimethoxyethane (DME) was used as the representative of LiPSs. The samples were mixed well with the Li_2_S_6_ solution and left undisturbed in the glovebox for 24 h. A clear loss in the yellow color was observed for all three samples, with the most evident change indicated by KL‐250‐800°C. This was further quantified by centrifuging the supernatant and performing UV–vis absorption spectroscopy, the results of which are depicted in Figure 3a. The inset shows a digital image of the adsorption test after 24 h. The UV−vis spectra of the pure Li_2_S_6_ sample contains two clear peaks at 350 nm and 420 nm represent the S_6_^2−^ and S_4_^2−^ anions [47]. The 800°C sample shows the strongest reduction in these peaks, compared to the other two samples, which show intermediate values, indicating that the optimal uptake was attained by carbonization at 800°C. The fibers sedimented at the end of this test were allowed to dry in the glovebox and their Raman spectra were obtained, which are shown in Figure 3b in the 100–2000 cm^−1^ range, covering both the polysulfide and the carbon peaks. For all the samples, clear polysulfide peaks are observed. The peaks at low Raman shifts of 130–230 cm^−1^ indicate the S_8_^2−^, the sharp peak at 417 cm^−1^ can be allocated to the S_4_^2−^ and S_6_^2−^ anions, that at 478 cm^−1^ to the S_3_^2−^ and S_4_^2−^ anions, that at 576 cm^−1^ to the S_5_^2−^ anion, and the peak at 748 cm^−1^ represents a combined peak for the different polysulfides (S_x_^2−^, x = 4–8) [48, 49]. This indicates clear adsorption of the LiPSs by all three samples. The amount of the polysulfides adsorbed can be further confirmed by the intensity of the D and G bands in the 1200−1700 cm^−1^ range. These bands are the clearest for the 600°C, with the intensity reducing for the 800°C sample, and nearly completely disappearing for the 1000°C sample. The gradual weakening of the carbon signals at higher carbonization temperatures may be related to stronger surface coverage by adsorbed polysulfide species and differences in surface structure. The UV–vis results are used to compare the overall LiPS adsorption ability, while Raman spectroscopy provides complementary evidence for the presence of surface‐bound polysulfide species.

Additionally, the XPS spectra for these three samples after Li_2_S_6_ adsorption were obtained, and the C *1s*, Li *1s*, and S *2p* spectra are shown in Figure S8a–c, respectively. The deconvoluted C *1s* peaks observed at 284.7, 285.2, 287.2, and 288.6 eV correspond to the sp^3^ carbons, weakly oxidized carbons, highly oxidized carbons, and carboxyl groups, respectively, and these peaks are consistent with those presented in Figure 2c [19]. The single clear peak at 56.1 eV in Figure S8b corresponds to the Li–S bond in the polysulfides, affording the first indication of the effective impregnation of the polysulfides within the CNF matrix for all three samples [19]. Finally, the deconvoluted S *2p* peaks are presented in Figure S8c. For all three samples, the doublet at 163.7 and 165.1 eV represents the bridging sulfur and that at 161.5 and 162.6 eV reflects the terminal sulfur in the adsorbed polysulfides [19]. For KL‐250‐600°C, no further species are resolved, indicating that the adsorbed polysulfide remains essentially intact and the interaction is confined to physisorption. This is consistent with its abundant but largely non‐conjugated surface functionalities that cannot mediate the charge transfer required for chemical anchoring. For KL‐250‐800°C, an additional doublet emerges at 167.0 and 168.3 eV, which can be attributed to thiosulfate species generated by surface‐mediated oxidation of the adsorbed polysulfide [50]. This provides direct evidence that chemisorption operates alongside physisorption. KL‐250‐1000°C also displays the thiosulfate doublet, while with a lower relative contribution. Two further features distinguish this sample. First, compared to the 600°C and 800°C samples, the terminal sulfur signal for the 1000°C sample shifts to higher binding energy, consistent with electron density being donated from terminal sulfur to Lewis‐acidic surface sites, which reflects a strong local interaction with the remaining polysulfide. Second, KL‐250‐1000°C exhibits the appearance of a new doublet at 161.0 and 162.3 eV, below the binding energy range of terminal sulfur and within that of sulfide (S^2−^). This is in line with the XRD patterns, in which the minor reflections associated with inorganic residues from the lignin precursor were most pronounced for this sample. This component is assigned to metal sulfides formed through the reaction of the polysulfide with ash‐derived inorganic residues exposed after high‐temperature carbonization [51, 52]. Because the number of such residual inorganic sites is finite, this pathway is inherently self‐limiting and does not translate into a higher overall polysulfide uptake.

Therefore, the S *2p* results describe a progression distinction. KL‐250‐600°C binds polysulfides only physically, KL‐250‐800°C combines physisorption with the most extensive surface‐mediated chemical conversion, and KL‐250‐1000°C, despite its higher graphitization and electrical conductivity, offers fewer polar anchoring sites so that its interaction with polysulfides is dominated by a limited number of localized, strongly reducing sites. The superior polysulfide affinity of KL‐250‐800°C inferred from these spectra is corroborated by the static adsorption test and quantification of the residual Li_2_S_6_ concentration via UV–vis absorption spectroscopy (Figure 3a).

Next, symmetric cells containing 10‐mm‐diameter free‐standing electrodes synthesized at three calcination temperatures were prepared to analyze the catalytic contribution of the fibers toward the sulfur reduction reaction. 20 µL of 0.41 M Li_2_S_6_ was used as the representative of the LiPSs and added to the electrolyte. The electrolyte used herein was 1 M bis(trifluoromethane)sulfonimide lithium salt (LiTFSI) in equal volumetric ratios of DOL and DME with 2 wt.% LiNO_3_ as the additive. The corresponding voltammograms obtained for the three samples attained at the sweep rate of 10 mV s^−1^ are shown in Figure 3c. The KL‐250‐600°C electrode exhibits featureless and weak redox responses, indicating its limited electrocatalytic activity toward polysulfide conversion. This could also be attributed to its poorer conductivity owing to lower carbonization temperatures. In contrast, distinct redox peak pairs are observed for the KL‐250‐800°C and KL‐250‐1000°C electrodes, confirming their enhanced catalytic capability for sulfur redox reactions. In the anodic scan, the a_1_, a_2_, and a_3_ peaks can be assigned to the stepwise oxidation of Li_2_S/Li_2_S_2_ to soluble polysulfides and ultimately to elemental sulfur, while the corresponding cathodic peaks c_1_, c_2_, and c_3_ reflect the reverse reduction process from sulfur species to lower‐order polysulfides and Li_2_S/Li_2_S_2_ [52, 53]. Notably, compared with the KL‐250‐1000°C electrode, the KL‐250‐800°C electrode displays sharper and more intense redox peaks, suggesting faster redox kinetics and more favorable electrocatalytic activity. These results demonstrate that carbonization at 800 °C provides the most effective structure for promoting polysulfide conversion and sulfur redox reversibility.

**FIGURE 4:**
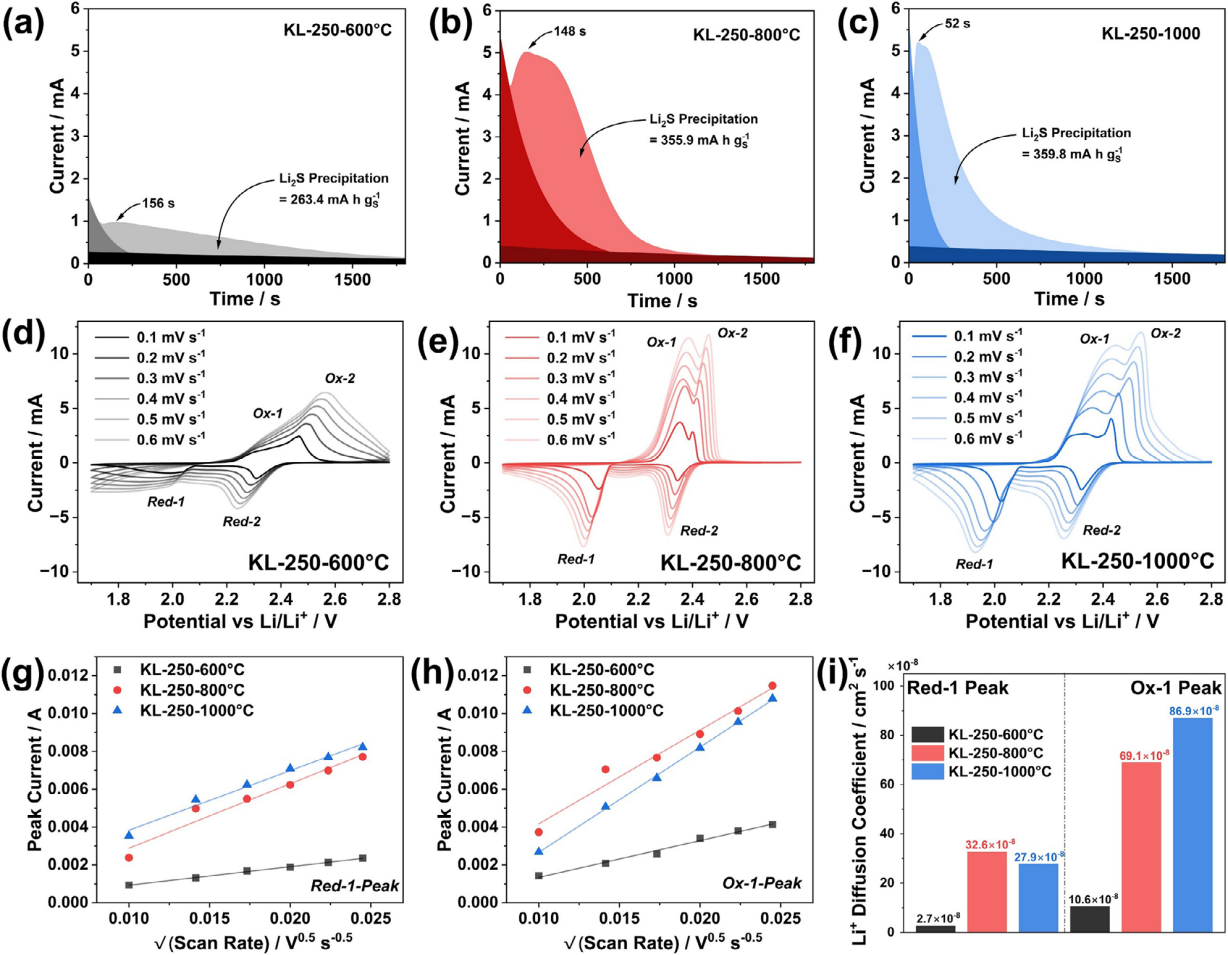
Li_2_S precipitation test for the (a) KL‐250‐600°C, (b) KL‐250‐800°C, and (c) KL‐250‐1000°C electrodes using Li_2_S_8_ as the catholyte, along with the calculated Li_2_S precipitation capacity. Cyclic voltammograms of the (d) KL‐250‐600°C, (e) KL‐250‐800°C, and (f) KL‐250‐1000°C free‐standing electrodes from 1.7 to 2.8 V measured at sweep rates of 0.1, 0.2, 0.3, 0.4, 0.5, and 0.6 mV s^−1^. Linear fitting of the peak current and the square root of the sweep rate based on the Randles–Ševčík equation for the (g) Red‐1 and (h) Ox‐1 peaks, as well as the correspondingly calculated (i) Li^+^ ion diffusion coefficients.

As the conversion of short‐chain polysulfides to Li_2_S theoretically affords 75% of the total capacity in Li–S batteries, the Li_2_S precipitation test was carried out using Li_2_S_8_ as the catholyte to study the sites available on the cathode for the deposition of the final reduction product [54]. The coin cells were assembled using the same electrodes as those for the symmetric cells, with 20 µL of 0.25 M Li_2_S_8_ added as the catholyte and 20 µL of the LiTFSI electrolyte as the anolyte. The progression of the current with time during this test for KL‐250‐600°C, KL‐250‐800°C, and KL‐250‐1000°C is shown in Figure 4a–c, respectively. The capacity arising from the reduction of Li_2_S_6_ and Li_2_S_8_ (darker colors in the plots) have been fitted and subtracted from the total capacity to obtain the Li_2_S precipitation capacity. The equations for fitted curves are listed in Table S2. The fastest deposition takes place for the 1000°C electrode with the peak of the Li_2_S precipitation curve occurring at 52 s, which also presents the highest precipitation capacity of 359.8 mAh g^−1^. The 800°C electrode requires longer time of 148 s but reaches a capacity of 355.9 mAh g^−1^, which is nearly as high as that of the 1000°C sample. Finally, the slowest deposition, starting at 156 s and lowest capacity of 263.4 mAh g^−1^ was presented by the 600°C electrode. This time for the peak current to be reached represents the point at which the Li_2_S nucleation rate is the highest and the onset of the growth regime for Li_2_S during precipitation. It can thus be concluded that the 1000°C electrode offers the highest number of sites of Li_2_S nucleation, with the nucleation also occurring the quickest. Although the 800°C electrode exhibits a nearly similar capacity, the nucleation is slower. The general trend among the three samples is similar to that followed by the fiber diameter observed from the SEM images in Figure 2. The thinner fibers offer higher surface‐to‐volume ratio as well as increased spaces between the fibers that act as the Li_2_S nucleation sites. These results indicate that higher calcination temperatures for lignin CNFs are beneficial to promote the liquid−solid transition during the sulfur reduction process.

To analyze the impact of the different carbonization temperatures on the internal impedance, coin cells with the free‐standing electrodes calcinated at different temperatures, using lithium metal as the anode, Li_2_S_8_ in DOL:DME (50:50 v:v) as the sulfur source, and 1 M LiTFSI in DOL:DME (50:50 v:v) with 2 wt.% LiNO_3_ as the electrolyte were assembled. The sulfur loading was 1.6 mg_S_ cm^−2^ and the electrolyte‐to‐sulfur (E/S) ratio was 30 µL_E_ mg_S_^−1^. One activation galvanostatic charge–discharge cycle was performed for cell activation. Subsequently, electrochemical impedance spectroscopy (EIS) was performed, and the characteristic Nyquist plots obtained are shown in Figure S9. The first resistive intercept at high frequencies corresponds to the electrolyte resistance and represents very low values here of 1–3 Ω for all electrodes. The first semi‐circle represents the total polysulfide charge transfer resistance from the bulk electrode and charge transfer at the electrode–electrolyte interface [55]. The second minor semi‐circle arises from the diffusion resistance in the solid–electrolyte interphase, and the final tail represents the polysulfide diffusion resistance in the electrolyte and separator [55, 56]. The total electrochemical impedance from coin cell assembled with the KL‐250‐800°C electrode is the lowest of 34.3 Ω, followed by the 1000°C and 600°C electrodes of 40.5 and 70.6 Ω. This indicates that the calcination temperature of 800°C offers the least hinderance to the Li^+^ and polysulfide ionic transfer. Subsequently, the cyclic voltammograms for the same coin cells were measured from 1.7 to 2.8 V at different sweep rates of 0.1, 0.2, 0.3, 0.4, 0.5, and 0.6 mV s^−1^ and the results are shown in Figure 4d–f. The peaks Red‐1 and Red‐2 represent the reduction of sulfur to long‐chain polysulfides and their subsequent conversion to Li_2_S_2_/Li_2_S as the final reduction products. The respective reversible oxidations of the same species are then indicated by the Ox‐1 and Ox‐2 peaks [6, 57]. For a better and direct comparison, the curves for the 0.1 and 0.6 mV s^−1^ for all electrodes are shown in Figure S10. The potential gap between Red‐1 and Ox‐1 peaks for the 800°C and 1000°C samples at 0.1 mV s^−1^ are 301.1 and 304.4 mV, respectively, indicating higher overpotential afforded by the 1000°C sample. The corresponding Red‐1 peak currents were noted to be 2.38 and 3.54 mA, as well as the Ox‐1 peak currents to be 3.74 and 2.68 mA, respectively. The corresponding potential gaps at the sweep rate of 0.6 mV s^−1^ are 381.3 and 513.9 mV, respectively, representing the lowest overpotential by the 800°C sample, with a significant increase for the 1000°C sample, highlighting its sluggish redox kinetics. Although the 1000°C sample demonstrates the highest Red‐1 peak current, the best redox reversibility was displayed by the 800°C sample. The Li^+^ ion diffusion coefficient was calculated using the Randles–Ševčík equation (at standard temperature and pressure conditions) for the Red‐1 and Ox‐1 peaks to quantify this [19, 58].

$$i_{p}=2.69\cdot n^{1.5}\cdot A\cdot C\cdot \sqrt{D_{Li^{+}}\cdot \nu }$$

where *i_p_* represents the peak current (A), *ν* is the sweep rate (V s^−1^), *n* is the number of electrons that participate in the reaction (here, 2), *A* is the electrode surface area (here 0.785 cm^2^), and C is the concentration of Li^+^ ions in the electrolyte (here 0.001 mol cm^−3^). A linear fit between *i_p_* and square root of the sweep rate is obtained by following the Randles–Ševčík equation, and these linear fits for the Red‐1 and Ox‐1 peaks are presented in Figure 4g,h, respectively. Based on the slope of this fitted straight line, the Li^+^ ion diffusion coefficient is calculated. The 600°C sample exhibits the lowest slope and the lowest peak current intensities for both the Red‐1 and Ox‐1 peaks. For the other two, the 1000°C sample presents the best performance during the reduction process, whereas the 800°C for the oxidation process. Regardless, for all samples, the Li^+^ diffusion coefficient values are higher in the charge process, indicating facilitation of the oxidative cycle (charge process) by the CNF morphology.

**FIGURE 5:**
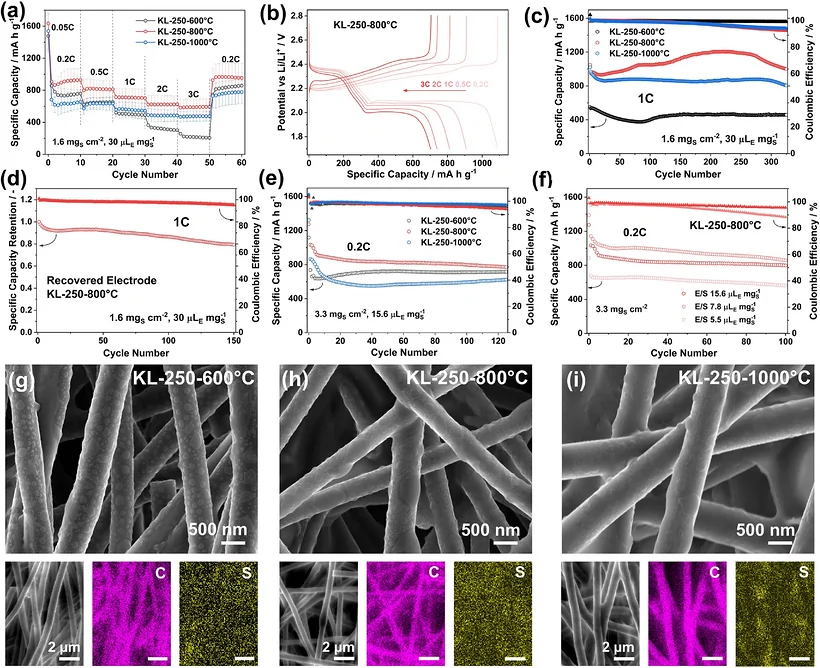
(a) Rate capability test for the KL‐250‐600°C, KL‐250‐800°C, and KL‐250‐1000°C free‐standing electrodes at 0.2 C, 0.5 C, 1 C, 2 C, 3 C, and back to 0.2 C for 10 cycles each. The values represent an average of three coin cells, with a first activation cycle of 0.05 C. (b) Galvanostatic charge–discharge (GCD) curves corresponding to the 0.2 C, 0.5 C, 1 C, 2 C, and 3 C rates for the KL‐250‐800°C electrode. (c) Long‐term cycling of the three electrodes at 1 C for 325 cycles. (d) Stable cycling of a recovered and reused KL‐250‐800°C electrode. (e) Stable cycling at 0.2 C at higher sulfur loading of 3.3 mg_s_ cm^−2^, with a lower electrolyte‐to‐sulfur (E/S) ratio of 15.6 µL mg_s_^−1^. (f) Comparison of cycling performance at different E/S ratios for the KL‐250‐800°C free‐standing electrode. SEM images of the (g) KL‐250‐600°C, (h) KL‐250‐800°C, and (i) KL‐250‐1000°C electrodes, disassembled in the fully charged state, with the corresponding energy‐dispersive X‐ray spectroscopy (EDX) mapping of carbon and sulfur.

The cycling performance and rate capability of the Li–S coin cells, which were assembled in same configuration as for CV measurements, were studied (1.6 mg_S_ cm^−2^, 30 µL_E_ mg_S_^−1^). The cells were cycled for 10 cycles each at different current densities of 0.2 C, 0.5 C, 1 C, 2 C, 3 C, and back to 0.2 C to analyze the rate performance, and the corresponding plot obtained is presented in Figure 5a. It must be noted that three coin cells were tested and the capacities shown here are an average of the three, with the standard deviations indicated via the error bars. The first cycle of 0.05 C was applied for cell activation. The KL‐250‐800°C electrode reveals the highest specific discharge capacity at all C‐rates, with the average discharge capacity delivered over the 10 cycles being 907, 814, 709, 623, 590, and 958 mAh g^−1^ at 0.2 C, 0.5 C, 1 C, 2 C, 3 C, and 0.2 C, respectively. This is additionally highlighted by the outstanding recovery when the current is changed from 3 C to 0.2 C. The KL‐250‐600°C electrode displays better capacity than the KL‐250‐1000°C electrode at a lower C‐rate of 0.2 C but demonstrates nearly similar values at 0.5 C and then drops significantly at higher C‐rates, would indicate that at higher C‐rates, the conductive nature of the electrode governs the capacity that can be obtained. The average capacities afforded by the KL‐250‐600°C electrode were 760, 645, 502, 322, 219, and 829 mAh g^−1^, and those by the KL‐250‐1000°C electrode were 638, 627, 556, 485, 478, and 760 mAh g^−1^ for the same current densities, respectively. This reveals that the carbonization temperature of 800°C is optimal for use as free‐standing electrodes, allowing increased sulfur utilization and improvement in the sulfur reduction kinetics. It can further be concluded that carbonization at higher temperatures provides stabilization and significant improvement in the conductivity, which is essential for providing stable cycling at high C‐rates. This is evidenced by the very low loss in capacity at high C‐rate for the electrode carbonized at 1000°C. The galvanostatic charge–discharge (GCD) curves for the KL‐250‐800°C electrode for each C‐rate are shown in Figure 5b, which shows the clear two‐plateau discharge curve. The first discharge plateau at the higher voltage of nearly 2.35 V corresponds to the Red‐2 peak labeled in Figure 4d, and it demonstrates the conversion of orthorhombic S_8_ to Li_2_S_4_, whereas the second plateau at the lower voltage around 2.05 V represents the Red‐1 peak, which corresponds to the further reduction of Li_2_S_4_ to the final reduction product Li_2_S [6, 57]. The plateaus for each C‐rate are flat and smooth, and with increasing C‐rate, the overpotential is observed to increase and the specific discharge capacity to decrease. For comparison, the GCD curves for the KL‐250‐600°C and KL‐250‐1000°C samples are shown in Figure S11a,b, respectively. The value of the overpotentials at different C‐rates for the coin cells assembled with electrodes carbonized at 600°C, 800°C, and 1000°C are listed in Tables S3–S5, respectively. It can be observed that at 0.2 C, the KL‐250‐1000°C electrode exhibits the lowest overpotential, with a similar value obtained at 0.5 C to the KL‐250‐800°C electrode. At higher C‐rates, the latter reveals the lowest overpotentials. Consequently, the KL‐250‐600°C demonstrates the highest overpotentials under all conditions. This indicates that the best charge transfer kinetics and diffusion kinetics are afforded by the 800°C sample, and the highest activation barriers are observed for the 600°C, the second reduction reaction (Li_2_S_4_ to Li_2_S) not reaching completion, which thereby has led to very low discharge capacity being obtained.

The long‐term stability of the coin cells was tested by cycling at 1 C over 325 cycles and the results obtained are shown in Figure 5c. The initial discharge capacities for the electrodes carbonized at 600°C, 800°C, and 1000°C are 549.4, 1048.4, and 1021.2 mAh g^−1^, respectively. After 325 cycles, the corresponding capacities are 458.3, 999.6, and 805.1 mAh g^−1^, indicating a capacity retention of 83.4%, 95.3%, and 78.8%, respectively. This highlights the excellent capacity retention ability of these electrodes, especially the KL‐250‐800°C electrode. Additionally, the capacity loss per cycle is recorded to be 0.055%, 0.013%, and 0.073%, respectively.

Furthermore, the sustainability of these electrodes was assessed by disassembling a short‐circuited coin cell, recovering the KL‐250‐800°C electrode and reassembling a new coin cell with a new lithium chip, and fresh addition of new sulfur. The cycling performance for this cell, prior to getting short‐circuited is depicted in Figure S12. Prior to coin cell assembly, the recovered electrode was washed several times with DME/DOL solution to remove any surface impurities and polysulfides left in the cathode. The cycling performance of the recovered electrode is shown in Figure 5d. Even during the second use, the cell portrays stable cycling, with 79.3% capacity retention after 150 cycles at 1 C, indicating outstanding recyclability and sustainability offered by this free‐standing electrode.

To further evaluate the stability of the electrode under more practical conditions, coin cells were assembled with a higher sulfur loading of 3.3 mg_S_ cm^−2^ and lower E/S ratio of 15.6 µL_E_ mg_S_^−1^, and the cycling performance at 0.2 C for 125 cycles is presented in Figure 5e. Even under these conditions, the same trend as that observed in the rate performance is noted, with the KL‐250‐800°C electrode offering the best performance over the cycling period. In the first 10 cycles, the KL‐250‐1000°C electrode affords higher capacities than the KL‐250‐600°C electrode, but once the cells stabilize at around 30 cycles, after SEI formation, sufficient electrode wetting, and complete sulfur utilization, including irreversible initial loss of LiPSs, the 1000°C electrode exhibits the poorest performance. The cells containing the KL‐250‐800°C electrode exhibit an average capacity loss of 0.23% per cycle over 125 cycles, whereas the 600°C and the 1000°C samples portray losses of 0.02% and 0.26% per cycle. Here, the lowest capacity loss is depicted by the 600°C sample, which is again due to its already low initial capacity.

Subsequently, the coin cell with the KL‐250‐800°C electrode was tested under even harsher conditions at E/S ratios of 7.8 and 5.5 µL_E_ mg_S_^−1^, and the cycling performance obtained is presented in Figure 5f. The initial capacities for the first 0.2 C cycle for the three E/S ratios of 15.6, 7.8, and 5.5 µL_E_ mg_S_^−1^ are observed to be 1033.4, 1142.0, and 885.0 mAh g^−1^, respectively. The corresponding capacities retained after 100 cycles have been noted to be 798.1, 859.2, and 561.1 mAh g^−1^, which represent capacity retentions of 77.2%, 75.2%, and 82.9%, respectively This emphasizes that even under extremely lean electrolyte conditions, the free‐standing electrodes can continue to cycle stably with excellent capacity retention. Moreover, the highest capacity was obtained for the E/S ratio of 7.8 µL_E_ mg_S_^−1^, which could be explained based on the presence of lesser amount of electrolyte, which restricts the dissolution of polysulfides and subsequently suppresses the shuttle effect.

Finally, post‐mortem analysis was conducted on the electrodes after cycling. The coin cells that were used for evaluating the rate performance were stopped in the fully charged state, which refers to the sulfur being present in the fully oxidized state of S_8_. The coin cells were disassembled, and the electrodes were recovered and allowed to dry inside the glovebox for a few minutes, which were then sealed and transported for SEM and energy‐dispersive X‐ray spectroscopy (EDX) measurements. The attained results for the KL‐250‐600°C, KL‐250‐800°C, and KL‐250‐1000°C electrodes are shown in Figure 5g–i, respectively. For all samples, it can be concluded that the free‐standing fiber structure of the electrodes was retained, with additional sulfur being deposited on and around the CNFs. The average diameter for all the samples is noted to increase after cycling, which can be explained based on the deposition of sulfur onto the fibers. The average diameters of the CNFs of the three electrodes are 602.7 ± 76.6, 525.8 ± 62.9, and 720.3 ± 97.4 nm, respectively. The corresponding Gaussian distributions are shown in Figure S13. The EDX images signify that sulfur particles are evenly distributed throughout the matrix around the fibers, supporting the claim that the increase in fiber size is attributed to the deposition of sulfur. The highest increase in diameter is observed for the 1000°C sample, which is in accordance with the Li_2_S precipitation experiment, indicating that the higher carbonization temperature assisted in providing more sites for the nucleation and growth, and subsequent sulfur retention. This would also highlight the better retention of the capacity offered by this electrode after each change in C‐rate.

Overall, these electrochemical results demonstrate that carbonization temperature critically regulates the sulfur host behavior of lignin‐derived CNFs, with insufficient carbonization at 600°C leading to sluggish redox kinetics, while excessive carbonization at 1000°C, leading to increase in amorphous carbon, improving specific conversion steps but compromising overall sulfur utilization. The 800°C electrode achieves the most balanced carbon structure for polysulfide confinement, sulfur redox conversion, and long‐term cycling stability, while its regenerability further highlights the sustainable and recyclable potential of the free‐standing cathode design.

## 3 Conclusion

In summary, lignin‐derived free‐standing CNF sulfur hosts were developed through electrospinning and controlled carbonization, demonstrating that carbonization engineering provides an effective route to optimize the polysulfide adsorption capability and sulfur redox conversion kinetics of the synthesized free‐standing electrodes. The optimized KL‐250‐800°C electrode delivered the best overall electrochemical performance, achieving a high discharge capacity of 1048.4 mAh g^−1^ at 1 C and retaining 999.6 mAh g^−1^ after 325 cycles, corresponding to a capacity retention of 95.3% and a low decay rate of 0.013% per cycle. It also maintained stable cycling under high‐sulfur‐loading and lean‐electrolyte conditions, retaining 561.1 mAh g^−1^ after 100 cycles at a sulfur loading of 3.3 mg cm^−2^ and an E/S ratio of 5.5 µL mg^−1^. Although the KL‐250‐1000°C electrode showed slightly enhanced Li_2_S deposition behavior and favorable oxidation kinetics, the KL‐250‐800°C electrode achieved the most balanced full‐cell performance. More importantly, the cycled recovered electrode could be reused after replenishing fresh Li_2_S_8_ catholyte, maintaining stable cycling with 79.3% capacity retention after 150 cycles at 1 C, confirming the structural robustness and regenerability of the free‐standing host. This work offers a sustainable, binder‐free, and recyclable electrode design strategy for high‐performance lithium–sulfur batteries.

## 4 Experimental Section

### 4.1 Materials and Reagents

Kraft lignin was obtained from Mercer Rosenthal (>59% dry content, 1.26% ash content). Anhydrous ethanol, bis(trifluoromethane)sulfonimide lithium salt (LiTFSI, anhydrous, 99.99%), lithium nitrate (LiNO_3_, 99.99%), 1,2‐dimethoxyethane (DME, anhydrous, 99.5%), 1,3‐dioxolane (DOL, anhydrous, 99.8%), sulfur powder, lithium sulfide (Li_2_S, 99.98%), polyvinyl pyrrolidone (PVP, M_w_ = 1 300 000), and N,N‐dimethylformamide (DMF, 99.8%) were purchased from Sigma–Aldrich. No additional treatments were done to the reagents before use.

### 4.2 Synthesis of Lignin Nanofibers via Electrospinning

Electrospinning was conducted using the Spinbox system from Bionica S. L. Paterna. Since lignin cannot be electrospun on its own, an additive co‐polymer was required to be able to obtain electrospun nanofibers. The electrospinning solution was prepared by adding 1.74 g of lignin, 0.46 g of PVP, and 5 mL DMF in a glass vial with a magnetic stirrer. The polymer amount in the solution was 32 wt.%. The solution was allowed to stir overnight inside a fume hood. Subsequently, this mixture was loaded into a 5‐mL syringe and connected to the flow rate controller of the Spinbox system. The distance between the needle and the aluminum collector was set to 15 cm, and a low flow rate of 1 mL h^−1^ was applied for conducting the electrospinning process with the voltage applied being ∼9 kV. After 5 h the lignin‐derived fibrous mat obtained was peeled off from the aluminum collector and was stabilized in air by heating at a very low rate of 0.5°C min^−1^ until 250°C for 1 h. This pre‐oxidation step prior to carbonization is essential to enhance cross‐linking of the fibers such that they do not merge or evaporate at high calcination temperatures. Next, the stabilized nanofiber mat was placed between two ceramic plates and inserted into the tube furnace. Carbonization was conducted at a heating rate of 5°C min^−1^ until the desired temperature of 600°C, 800°C, or 1000°C was reached and held for 2 h under argon flow in a horizontal tube furnace. Afterward, the furnace was allowed to naturally cool to room temperature. This afforded the free‐standing electrode with a thickness of ∼300 nm, which was directly cut into 10‐mm discs and employed as binder‐free electrodes in coin cells.

### 4.3 Li_2_S_6_ Adsorption Test

To observe the adsorptive capability of the synthesized free‐standing electrodes to adsorb polysulfides, an adsorptive test was conducted with Li_2_S_6_ being employed as a LiPS representative. The 0.1 M Li_2_S_6_ stock solution was prepared by dissolving sulfur and Li_2_S in 5:1 molar ratio in equal volumetric parts of 1,3‐dioxolane (DOL) and dimethyl ether (DME) solvents (50:50, v:v) and vigorously stirring at 80°C for 48 h. This was then diluted to 2 mM in DOL:DME (50:50, v:v). The fibers calcinated at different temperatures were cut into small pieces and 20 mg of each was placed in small glass vials. The 2 mM Li_2_S_6_ solution was added to each of these vials, which were shaken vigorously for complete dispersion within the solution and left undisturbed for 24 h until the fibers sedimented. Considering the air‐sensitive nature of this test, all the above steps were carried out inside an argon‐filled glovebox. The supernatant was centrifuged and their UV–vis spectra were obtained to quantify the degree of change in the yellow color. The precipitated fibers in the vials were allowed to dry overnight, after which Raman spectra were obtained for them.

### 4.4 Material Characterization

The morphology of the lignin‐derived fibers obtained at the different stages of the free‐standing electrode synthesis process was studied using scanning electron microscopy (SEM) using the Zeiss Gemini electron microscope, at an accelerating voltage of 5 kV. The fibers were cut and directly stuck onto the carbon tape attached to the sample holder for SEM imaging. For post‐mortem SEM and energy‐dispersive X‐ray spectroscopy (EDX) imaging of the electrodes after cycling, this was done inside the glovebox, wherein the coin cells were disassembled and the electrode was recovered, washed with DOL, cut, and stuck onto the holder and sealed for transport and analyses. Transmission electron microscopy (TEM) measurements were carried out using a JEOL JEM‐2100 microscope (JEOL GmbH, Eching, Germany) operated at an acceleration voltage of 200 kV and equipped with a 4k × 4k CMOS digital camera (TVIPS TemCam‐F416). For sample preparation, the materials were sonicated in methanol for 5 min, after which 5 µL of the resulting dispersion was deposited onto Lacey carbon‐coated copper TEM grids (200 mesh, Science Services). The grids were subsequently dried under a fume hood. The Autosorb‐1 gas sorption analyzer by Quantachrome Instruments was implemented for obtaining the N_2_ adsorption–desorption isotherms and Brunauer–Emmett–Teller (BET) specific surface areas via the multipoint analysis in the 0–0.3relative pressure range. Netzsch TG 209 F1 Iris thermal analyzer was applied for performing thermogravimetric analysis (TGA). The measurement was carried out under an inert argon atmosphere from room temperature to 800°C at a linear heating ramp of 10°C min^−1^. The Agilent 8453 Spectrophotometer was employed for carrying out ultraviolet–visible (UV–vis) absorption spectroscopy to quantify the difference in the intensity of the yellow color of the supernatant after the Li_2_S_6_ adsorption test. The supernatants were centrifuged to ensure that no nanofibers were present in the cuvettes during measurement. High‐resolution X‐ray photoelectron spectroscopy (XPS) was conducted by using a K‐alpha spectrometer from Thermo Fischer Scientific on carbonized fibers. The step size for obtaining high‐resolution spectra was 0.05 eV. Raman spectra were acquired on a Thermo Fisher Scientific DXR3 Raman microscope using a 532 nm laser with an excitation power of 0.5 mW. Sample preparation was done in an Ar‐filled glovebox. Air‐sensitive samples were sealed in an ECC‐Opto‐10 cell equipped with a sapphire window from EL‐CELL.

### 4.5 Electrochemical Testing

The coin cells were assembled in an argon‐filled glovebox using the 2025 coin cell components. 1 M LiTFSI in equal volumetric ratios of DOL and DME with 2 wt.% of LiNO_3_ as the additive was used as the electrolyte. Celgard 2400 was cut into 16‐mm discs and used as the separator and 12‐mm thick lithium metal disc was employed as the anode. When assembling symmetric cells, 20 µL of 0.1 M Li_2_S_6_ was used as the catholyte (on the cathode side of the separator) and 20 µL of the electrolyte as the anolyte (on the anode side of the separator). For Li–S cells utilized for cycling cyclic voltammetry (CV) and galvanostatic charge–discharge (GCD) measurements, sulfur loading was done using 5 µL of 1 M Li_2_S_8_ in equal volumetric parts of DOL and DME, which translates to a sulfur loading of 1.6 mg_S_ cm^−2^. For a higher sulfur loading of 3.3 mg_S_ cm^−2^, 10 µL of the same 1 M Li_2_S_8_ solution was added instead. 40 µL of the LiTFSI electrolyte was used for the coin cells (20 µL on each side of the separator). The corresponding E/S ratios are 30 and 13 µL_E_ mg_S_^−1^. For the other E/S ratios, 10 µL of 1 M Li_2_S_8_ was always used with different amounts of electrolyte added in the amounts of 40, 20, and 14 µL corresponding to E/S ratios of 15.6, 7.8, and 5.5 µL_E_ mg_S_^−1^.

The Biologic VMP3 multichannel potentiostat was implemented for perform cyclic voltammetry (CV) at sweep rates of 0.1, 0.2, 0.3, 0.4, 0.5, and 0.6 mV s^−1^ in the voltage range of 1.7 V to 2.8 V vs. Li^+^/Li. The Neware battery testing system was used to perform galvanostatic charge–discharge (GCD) measurements in the voltage range of 1.7–2.8 V vs. Li^+^/Li. The rate performance was measured at 0.2 C, 0.5 C, 1 C, 2 C, 3 C, and back to 0.2 C for 10 cycles each. The long‐term cycling performance of the free‐standing electrodes was tested at 1 C over 400 cycles. It must be noted that for all GCD measurements, one 0.05 C cycle for cell activation was performed. The symmetric cell CV were obtained in the potential window from −0.8 to 0.8 V at 10 mV s^−1^. The GAMRY Interface 1010E potentiostat was implemented to perform potentiostatic electrochemical impedance spectroscopy (EIS) in the frequency range of 1 000 000 to 0.01 Hz, which was performed after coin cell activation.

### 4.6 Li_2_S Precipitation Test

Sulfur and Li_2_S were mixed in 7:1 in molar ratio in DOL:DME solvent (50:50, v:v) and stirred vigorously for 48 h at 80°C to prepare the Li_2_S_8_ solution. Coin cells were assembled using 20 µL of 0.25 M Li_2_S_8_ as the catholyte and the LiTFSI electrolyte as the anolyte. The cells were discharged galvanostatically at 0.05 C to 2.16 V and held potentiostatically at 2.05 V for Li_2_S nucleation and growth for 15 h.

## Funding

Carl–Zeiss‐Foundation via the Durchbrüche‐Programm (project Upgrading lignin for fair raw materials – LignUp)

## Conflicts of Interest

The authors declare no conflicts of interest.

## Supporting information




**Supporting File**: gch270157‐sup‐0001‐SuppMat.docx.

## Data Availability

The data that support the findings of this study are available from the corresponding author upon reasonable request.
