# Supplementary material for: Reusable Lignin‐Derived Free‐Standing Carbon Nanofiber Sulfur Hosts via Carbonization Engineering for Sustainable Lithium–Sulfur Batteries
Source: Glob Chall. 2026 Sep 8;10(9):e70157. doi: 10.1002/gch2.70157 (PMC13554718; doi:10.1002/gch2.70157)
Supplement: Supplementary file 1 [file GCH2-10-e70157-s001.docx]

Supporting Information

Reusable Lignin-Derived Free-Standing Carbon Nanofiber Sulfur Hosts *via* Carbonization Engineering for Sustainable Lithium–Sulfur Batteries

Kshitij Mahesh Kakad, Qingping Wu*, Seyedrashid Mirmasoomi, Sijia Cao, Johannes Schenk, Marius Hermesdorf, Vikram Raghuraman, Liqiang Lu, Martin Oschatz, Yan Lu*

**Figure S1.** Digital images of the (a) as-prepared electrospun lignin carbon nanofibers and (b) the subsequently pre-oxidized fibrous mat.

**Figure S2.** Low-resolution SEM images of the (a) as-prepared electrospun lignin CNFs and (b) stabilized CNFs, as well as after carbonization at (c) 600°C, (d) 800°C, and (e) 1000°C.

**Figure S3.** Gaussian distribution of the carbonized lignin CNFs obtained from measuring 150 fibers from low-resolution SEM images for the (a) KL-250-600°C, (b) KL-250-800°C, and (c) KL-250-1000°C samples.

**Figure S4.** Fourier-transform infrared spectra of the as-prepared and pre-oxidized electrospun lignin CNFs as well as the carbonized fibers at 600°C, 800°C, and 1000°C.

**Figure S5.** Low-resolution transmission electron microscopy images of the electrospun lignin CNFs after carbonization at (a) 600°C, (b) 800°C, and (c) 1000°C.

**Figure S6.** (a) Pore size distributions and (b) cumulative pore volumes of the lignin CNFs carbonized at different temperatures.

**Figure S7.** Gaussian fits of the D1, D2, G, and A bands of the Raman spectra the (a) KL-250-600°C, (b) KL-250-800°C, and (c) KL-250-1000°C samples.

**Figure S8.** (a) C *1s*, (b) Li *1s*, and (c) S *2p* XPS spectra for KL-250-600°C, KL-250-800°C, and KL-250-100°C after Li_2_S_6_ adsorption.

**Figure S9.** Nyquist plot for the Li–S coin cells assembled with the KL-250-600°C, KL-250-800°C, and KL-250-1000°C electrodes after the first activation cycle.

**Figure S10.** Cyclic voltammograms of the KL-250-600°C, KL-250-800°C, and KL-250-1000°C free-standing electrodes from 1.7 to 2.8 V measured at the sweep rates of (a) 0.1 mV s^−1^ and (b) 0.6 mV s^−1^.

**Figure S11.** Galvanostatic charge–discharge (GCD) curves corresponding to the 0.2C, 0.5C, 1C, 2C, and 3C rates for the (a) KL-250-600°C and (b) KL-250-1000°C electrodes.

**Figure S12.** Cycling performance at 1C of a coin cell that short-circuited after 150 cycles. The cell was disassembled, and the electrode was recovered, washed, and reused in a new coin cell.

**Figure S13.** Gaussian distribution of the carbonized lignin-derived CNFs obtained from measuring 150 fibers from low-resolution SEM images for the (a) KL-250-600°C, (b) KL-250-800°C, and (c) KL-250-1000°C samples after they are used in coin cells at the end of the rate performance measurements.

**Table S1.** Elemental analysis of the synthesized lignin fibers at different stages of the preparation of the free-standing electrodes.

| Element | As-KL / wt.% | KL-250°C / wt.% | KL-250-600°C / wt.% | KL-250-800°C / wt.% | KL-250-1000°C / wt.% |
| --- | --- | --- | --- | --- | --- |
| C | 60.76 | 61.41 | 84.75 | 83.49 | 88.85 |
| H | 6.17 | 5.15 | 2.03 | 0.76 | 0.39 |
| N | 3.49 | 3.64 | 1.97 | 1.81 | 2.18 |
| S | 1.35 | 0.79 | 0.31 | 0.61 | 0.61 |
| Sum | 71.77 | 70.99 | 89.06 | 86.67 | 92.03 |
| Remaining | 28.23 | 29.01 | 10.94 | 13.33 | 7.97 |

**Table S2.** Fitted curves for the reduction of Li_2_S_6_ and Li_2_S_8_ in the precipitation test.

| Electrode | Li_2_S_6_ | Li_2_S_8_ | Area under the Curve / mA s | Li_2_S precipitation / mAh g_S_^−1^ |
| --- | --- | --- | --- | --- |
| KL-250-600°C | $0.267\cdot{exp(-5.04\times10}^{-4})$ | $1.548\cdot{exp(-8.03\times10}^{-3})$ | 1213.9 | 263.4 |
| KL-250-800°C | $0.392\cdot{exp(-6.84\times10}^{-4})$ | $5.367\cdot{exp(-4.86\times10}^{-3})$ | 1547.6 | 355.9 |
| KL-250-1000°C | $0.369\cdot{exp(-3.95\times10}^{-4})$ | $5.583\cdot{exp(-12.19\times10}^{-3})$ | 1920.3 | 359.8 |

**Table S3.** The discharge and charge voltages from the GCD curve for C-rate in the rate capability test from **Figure S7a** for the KL-250-600°C electrode, as well as the respective overpotentials. These potentials are taken at the half value of the total discharge capacity.

| C-Rate | Discharge Voltage / V | Charge Voltage / V | Overpotential / V |
| --- | --- | --- | --- |
| 0.2C | 2.076 | 2.301 | 0.225 |
| 0.5C | 2.039 | 2.339 | 0.300 |
| 1C | 2.005 | 2.397 | 0.392 |
| 2C | 2.003 | 2.475 | 0.472 |
| 3C | 1.964 | 2.539 | 0.575 |

**Table S4.** The discharge and charge voltages from the GCD curve for C-rate in the rate capability test from **Figure 5b** for the KL-250-800°C electrode, as well as the respective overpotentials. These potentials are taken at the half value of the total discharge capacity.

| C-Rate | Discharge Voltage / V | Charge Voltage / V | Overpotential / V |
| --- | --- | --- | --- |
| 0.2C | 2.094 | 2.287 | 0.193 |
| 0.5C | 2.079 | 2.299 | 0.220 |
| 1C | 2.0599 | 2.314 | 0.254 |
| 2C | 2.028 | 2.337 | 0.309 |
| 3C | 1.998 | 2.353 | 0.355 |

**Table S5.** The discharge and charge voltages from the GCD curve for C-rate in the rate capability test from **Figure S7b** for the KL-250-1000°C electrode, as well as the respective overpotentials. These potentials are taken at the half value of the total discharge capacity.

| C-Rate | Discharge Voltage / V | Charge Voltage / V | Overpotential / V |
| --- | --- | --- | --- |
| 0.2C | 2.093 | 2.265 | 0.172 |
| 0.5C | 2.069 | 2.289 | 0.220 |
| 1C | 2.051 | 2.325 | 0.274 |
| 2C | 1.999 | 2.331 | 0.332 |
| 3C | 1.938 | 2.435 | 0.497 |
